# Randomized, placebo-controlled, single-blind phase 1 studies of the safety, tolerability, and pharmacokinetics of BRII-196 and BRII-198, SARS-CoV-2 spike-targeting monoclonal antibodies with an extended half-life in healthy adults

**DOI:** 10.3389/fphar.2022.983505

**Published:** 2022-09-06

**Authors:** Xiaohua Hao, Zheng Zhang, Ji Ma, Lin Cheng, Yun Ji, Yang Liu, Dong Zhao, Wen Zhang, Chunming Li, Li Yan, David Margolis, Qing Zhu, Yao Zhang, Fujie Zhang

**Affiliations:** ^1^ Beijing Ditan Hospital, Capital Medical University, Beijing, China; ^2^ Institute for Hepatology, National Clinical Research Center for Infectious Disease, Shenzhen Third People’s Hospital, The Second Affiliated Hospital, School of Medicine, Southern University of Science and Technology, Shenzhen, Guangdong, China; ^3^ Brii Biosciences Inc, Durham, NC, United States; ^4^ TSB Therapeutics, Beijing, China

**Keywords:** SARS-CoV-2, monoclonal antibody, extended half-life, safety, pharmacokinetics, neutralizing activity

## Abstract

**Background:** BRII-196 and BRII-198 are two anti-SARS-CoV-2 monoclonal neutralizing antibodies as a cocktail therapy for treating COVID-19 with a modified Fc region that extends half-life.

**Methods:** Safety, tolerability, pharmacokinetics, and immunogenicity of BRII-196 and BRII-198 were investigated in first-in-human, placebo-controlled, single ascending dose phase 1 studies in healthy adults. 44 participants received a single intravenous infusion of single BRII-196 or BRII-198 up to 3,000 mg, or BRII-196 and BRII-198 combination up to 1500/1500 mg, or placebo and were followed up for 180 days. Primary endpoints were incidence of adverse events (AEs) and changes from pre-dose baseline in clinical assessments. Secondary endpoints included pharmacokinetics profiles of BRII-196/BRII-198 and detection of anti-drug antibodies (ADAs). Plasma neutralization activities against SARS-CoV-2 Delta live virus in comparison to post-vaccination plasma were evaluated as exploratory endpoints.

**Results:** All infusions were well-tolerated without systemic or local infusion reactions, dose-limiting AEs, serious AEs, or deaths. Most treatment-emergent AEs were isolated asymptomatic laboratory abnormalities of grade 1-2 in severity. BRII-196 and BRII-198 displayed pharmacokinetics characteristic of Fc-engineered human IgG1 with mean terminal half-lives of 44.6–48.6 days and 72.2–83.0 days, respectively, with no evidence of interaction or significant anti-drug antibody development. Neutralizing activities against the live virus of the SARS-CoV-2 Delta variant were maintained in plasma samples taken on day 180 post-infusion.

**Conclusion:** BRII-196 and BRII-198 are safe, well-tolerated, and suitable therapeutic or prophylactic options for SARS-CoV-2 infection.

**Clinical Trial Registration:** ClinicalTrials.gov under identifiers NCT04479631, NCT04479644, and NCT04691180.

## Introduction

As of June 2022, over 530 million people worldwide had been infected by SARS-CoV-2, resulting in more than six million deaths by coronavirus disease 2019 (COVID-19)^1^. The number of cases continues to rise, indicating remaining gaps for treatment and prevention to address. Neutralizing monoclonal antibodies (mAbs) represent a promising option for preventing and treating known and emerging infectious diseases, including viral infections (Jin et al., 2017). Clinical trials of mAbs have reported clinical benefits for treatment and prevention (Chen et al., 2021; Weinreich et al., 2021), which led to the emergent use authorization approval of several mAbs globally.

BRII-196 and BRII-198 are two recombinant human IgG1 mAbs derived directly from human B cells of patients who recovered from COVID-19 (Ju et al., 2020). Targeting distinct epitope regions in RBD in coronavirus spike glycoproteins non-competitively helps BRII-196 and BRII-198 to retain neutralizing activities against the original isolate of SARS-CoV-2 and major SARS-CoV-2 variants of concern, including.1.1.7 (Alpha), B.1.351 (Beta), P.1 (Gamma), B.1.429 (Epsilon), B.1.617.2 (Delta), AY.4.2 (Delta Plus), C.37 (Lambda), B.1.621 (Mu), B.1.1.529-BA.1 (Omicron), and BA.2 (Omicron subvariants) (Ji et al. submitted; Wang et al., 2021a; Wang et al., 2021b; Wang et al., 2022). In addition, BRII-196 and BRII-198 are engineered with a triple-amino-acid (M252Y/S254T/T256E [YTE]) substitution in the fragment crystallizable (Fc) region to allow for an extended half-life (Robbie et al., 2013; Saunders, 2019). The long-acting feature positions BRII-196/BRII-198 as a promising pre-exposure prevention option among the population who are either contraindicated or could not mount sufficient immunity against SARS-COV-2 after vaccination.

We designed and conducted the first-in-human phase 1 studies in healthy adult participants to evaluate the safety, tolerability, and pharmacokinetics (PK) of BRII-196 and BRII-198 individually and in combination, and plasma neutralizing activities associated with the prolonged effect of this antibody combination.

## Materials and methods

### Study design and participants

BRII-196-001, BRII-198-001, and BRII-196-198-001 are first-in-human phase 1, randomized, single-blind, placebo-controlled, single ascending dose studies in which BRII-196 and BRII-198 were evaluated separately and in combination among healthy adults. These studies were conducted at a single phase 1 unit in China from July 2020 to September 2021 (registered at ClinicalTrials.gov under registration numbers NCT04479631, NCT04479644, and NCT04691180). The study protocols, amendments, and informed consent forms were reviewed and approved by the Ethical Committee at the site (numbers of authorization in Section S1 in the Supplementary Appendix A1). The studies were conducted in accordance with the International Council for Harmonisation of Technical Requirements for Pharmaceuticals for Human Use (ICH) Guidance for Good Clinical Practice guidelines and all applicable local regulatory requirements and laws. Participants provided written informed consent before any study-related procedures were performed https://coronavirus.jhu.edu/map.html.

Eligible participants were healthy males or females who were aged 18–49 years, had a body weight ≤100 kg and a body mass index of 18-24 kg/m^2^, and were in good health determined as no clinically significant findings from medical history or physical examination including vital signs, electrocardiogram (ECG), and clinical laboratory assessments. Women were not pregnant or lactating. Exclusion criteria included a history of SARS-CoV-2 infection or exposure, a history of COVID-19 vaccination, a history of severe allergic reactions, use of any medications that were started within 14 days before randomization, a positive test for human immunodeficiency virus (HIV) or hepatitis B virus or hepatitis C virus, a history of drug or alcohol abuse within 1 year before screening, or any history of a medical or psychiatric condition that would place them at risk or interfere with study participation.

### Study procedures

Three dose levels (750, 1500, and 3,000 mg) were evaluated for each mAb separately, and two dose levels (750/750 mg and 1500/1500 mg) were evaluated for the mAb combination, initiated in a dose-escalation design. In each cohort, participants were randomized at a 3:1 ratio to receive either the mAb(s) or placebo. BRII-196, BRII-198, or placebo was dispensed into normal saline and was administered *via* intravenous infusion on day 1. The infusion was administered at a maximum rate of 4 ml/min. The participants were masked to the treatment assignment and were admitted to the phase 1 unit for monitoring for 7 days post-dosing. A Safety Review Committee made dose-escalation recommendations upon review of predefined safety data.

### Study endpoints

Primary endpoints were the incidence of AEs and change from pre-dose baseline in clinical assessments, including vital signs, ECG readings, and laboratory results. Secondary endpoints included the pharmacokinetics profiles and presence of ADAs to BRII-196/BRII-198 in samples collected after dosing for up to 180 days. Exploratory endpoints were neutralization activities against live SARS-CoV-2 virus of Delta variant using plasma samples taken up to 6 months after the administration of BRII-196 and BRII-198 combination.

### Safety assessments

Vital signs, physical examinations, safety laboratory tests including hematology and chemistry, and ECG were obtained before and after study drug administration on days 2, 4, 6, 8, 15, 22, 31, 61, 91, 121, and 151, and at the end of the study on day 181. All AEs, including infusion reactions and SAEs occurring throughout the trial, were recorded and assessed by study physicians at the site. AEs were coded with the Medical Dictionary for Regulatory Activities (MedDRA) version 23.1, and severity was graded using the Common Terminology Criteria for Adverse Events (CTCAE) version 5.0, 27 November 2017.

### Pharmacokinetics and immunogenicity assessments

The pharmacokinetics parameters were evaluated using blood samples collected before dosing, at 3 and 8 h post-dose, and on days 2, 4, 6, 8, 15, 22, 31, 61, 91, 121, 151, and 181. BRII-196 or BRII-198 serum concentrations were measured using validated enzyme-linked immunosorbent assays (ELISA) with the lower limit of quantitation (LLOQ) at 150 ng/ml. Serum samples for anti-drug antibody (ADA) assays were collected at the following time points: pre-dose on day 1 and post-dose on days 15, 31, and 181. Two validated titer-based 3-stage ELISA assays were used to detect the presence of ADAs from participants over the study durations.

### Neutralization of live virus delta variant

The measurement of neutralization was based on a published method (Ju et al., 2020). The variant B.1.617.2 is a clinical isolate (SZTH012 strain, Accession No. GWHBFWZ01000000 at the Genome Warehouse in National Genomics Data Center) containing 9 mutations, including T19R, G142D, 156-157del, R158G, L452R, T478K, D614G, P681R, and D950N. Serially diluted plasma samples (by a factor of 3), starting at 20-fold dilution, were incubated with an equal volume of live virus at 37°C for 1h. Mixtures were then transferred to 96-well plates seeded with Vero E6 cells and allowed to absorb for 1 h at 37°C. Inoculums were then removed before adding the overlay media (100 μl opti-MEM containing 1.6% Carboxymethylcellulose, CMC). The plates were then incubated at 37 °C for 24 h. Next, overlays were removed, and cells were fixed with 4% paraformaldehyde solution for 30 min. Cells were permeabilized with 0.2% Triton X-100, washed with PBS twice, and incubated with HRP-conjugated SARS-CoV-2 NP mAb P301-F7 for 2 h at room temperature (Zhou et al., 2022). The reactions were developed with KPL TrueBlue Peroxidase substrates (Seracare Life Sciences Inc.). The numbers of SARS-CoV-2 foci were calculated using an ELISpot reader (Cellular Technology Ltd.). All diluted samples were measured in duplicate. A standard positive control sample was used to ensure the comparability of experiments. Samples used for evaluation were plasma samples collected from six participants who received BRII-196/BRII-198 combination before dosing, at 3 h post-dose, and on days 91 and 181. In addition, from the vaccine (BBIBP-CorV inactivated vaccine) follow-up group (∼130 people), 10 participants with high, medium, and low antibody levels representing 90% of the follow-up population at 7 months post second vaccination were selected. Neutralizing activity of these 10-pair of plasma at 7 months post second vaccination and 2 weeks post third vaccination were tested. For a given sample, the 50% inhibitory dilution (ID50) was calculated through log (inhibitor) vs. normalized response variable slope (four parameters) modeling, where a greater ID50 value is associated with higher neutralization activity.

### Statistical analysis

The sample size of each study was consistent with a phase 1 first-in-human study. The safety analysis included all participants who were randomized and received any dose of the study drug. Categorical and continuous data were summarized descriptively. Participants were analyzed according to the study drug they received. Participants receiving placebo in different cohorts of each study were pooled in the analysis as appropriate.

The PK parameters were estimated by non-compartmental analyses using WinNonlin module in the Phoenix Platform (version 8.3.1.5014, Certara Inc., Princeton, NJ 08540). Calculations were performed prior to rounding, and nominal sampling times were used in the pharmacokinetic analysis. All pharmacokinetic parameters and summary statistics are reported to three significant digits except for T_max_ (reported in median values), which is reported to 1 decimal place.

For the assessment of plasma neutralization activity, the 50% inhibitory dilution (ID_50_) was calculated using GraphPad Prism 8.0 software by log(inhibitor) vs. normalized response variable slope (four parameters) model. Results were displayed descriptively.

## Results

45 participants were randomized, with 16, 17, and 12 participants enrolled into single BRII-196 evaluation (BRII-196-001 study), single BRII-198 evaluation (BRII-198-001 study), and mAb combination evaluation (BRII-196-198-001 study), respectively (Study participant flow diagrams in Supplementary Figures S1–S3). The demographics of the study population are summarized in Table 1. The majority of the participants were male (37/45, 82.2%). The median age of treatment groups ranged between 22 and 37.5 years. One randomized participant withdrew before dosing.

**TABLE 1:** Participant demographics and baseline characteristics.

	BRII-196-001				BRII-198-001				BRII-196-198-001		
	750 mg (n = 3)	1500 mg (n = 6)	3,000 mg (n = 3)	Placebo (n = 4)	750 mg (n = 3)	1500 mg (n = 7)	3,000 mg (n = 3)	Placebo (n = 4)	750/750 mg (n = 3)	1500/1500 mg (n = 6)	Placebo (n = 3)
Median age, years	22.0	32.5	34.0	33.5	30.0	33.0	30.0	37.5	25.0	24.0	27.0
Men	3 (100%)	4 (67%)	3 (100%)	3 (75%)	2 (67%)	7 (100%)	3 (100%)	3 (75%)	2 (67%)	5 (83%)	2 (67%)
Women	0	2 (33%)	0	1 (25%)	1 (33%)	0	0	1 (25%)	1 (33%)	1 (17%)	1 (33%)
Race											
Han, Chinese	3 (100%)	6 (100%)	2 (67%)	3 (75%)	3 (100%)	6 (86%)	3 (100%)	4 (100%)	3 (100%)	5 (83%)	3 (100%)
Other, Chinese	0	0	1 (33%)	1 (25%)	0	1 (14%)	0	0	0	1 (17%)	0 (0%)
Median body-mass index, kg/m^2^	22.80	21.15	21.60	22.90	23.70	21.00	22.30	23.10	22.30	21.75	20.40

A total of 12 participants received a single dose of BRII-196 intravenous (IV) infusion, 12 participants received a single dose of BRII-198 IV infusion, and 9 participants received BRII-196 and BRII-198 administered IV sequentially. Eight participants received placebo (normal saline). One participant randomized in BRII-198-001 study did not receive the infusion because of elevated blood pressure before dosing. All participants received their full planned doses except for three participants assigned to 750 mg of BRII-196 and 1 participant assigned to placebo where the actual volume administered was approximately 10% less than the intended volume due to the remaining volume left within the infusion set.

### Safety and tolerability

AEs reported are summarized in Tables 2, 3, 4. BRII-196 and BRII-198 separately or in combination were well-tolerated. No deaths, serious adverse events (SAEs), treatment-emergent adverse events (TEAEs) leading to early termination, or infusion-related reactions occurred during the study. Overall, thirty-six (82%) participants had at least one TEAE, including 26 (79%) out of 33 participants receiving BRII-196 and/or BRII-198 compared to 10 (90%) out of 11 participants receiving placebo. The most common TEAEs were isolated asymptomatic laboratory abnormalities of CTCAE grade 1 or 2 in severity that did not require medical intervention and typically were normalized or returned to baseline levels within 4 weeks (Supplementary Table S1, S2). Eight out of 33 participants had 11 grade 1 or 2 AEs that were considered by the investigators as related to BRII-196 or BRII-198, including decreased white blood cell count, decreased neutrophil count, decreased lymphocyte count, increased blood total bilirubin, and increased alanine aminotransferase (Supplementary Table S3, S4). Bilirubin elevation and alanine aminotransferase elevation were separate events that occurred in different subjects. No dose-dependent pattern of any AEs was observed.

**TABLE 2:** Summary of adverse events of BRII-196-001 study.

	BRII-196 750 mg (n = 3)	BRII-196 1500 mg (n = 6)	BRII-196 3,000 mg (n = 3)	Placebo (n = 4)
Participants with any TEAE	3 (100%)	5 (83%)	2 (67%)	4 (100%)
Participants with Grade 1 TEAE	3 (100%)	5 (83%)	2 (67%)	4 (100%)
Participants with Grade 2 TEAE	1 (33%)	3 (50%)	0	2 (50%)
Participants with Grade 3 or above TEAE	0	0	0	0
Participants with serious TEAE	0	0	0	0
Participants with infusion reaction TEAE	0	0	0	0
Participants with TEAEs related to study drug	1 (33%)	3 (50%)	0	0
Participants with any TEAE leading to discontinuation of study drug	0	0	0	0
Death	0	0	0	0
Number of participants with AEs by MedDRA SOC^a^				
Investigations	3 (100%)	4 (67%)	2 (67%)	4 (100%)
Infections and infestations	1 (33%)	0	0	2 (50%)

Abbreviations: TEAE, treatment-emergent adverse event; SOC, system organ class. Participants who experienced the same AE on more than one occasion (based on the specific category) are counted once in each relevant category. Percentages are based on the number of participants in the treatment group.

a Only SOCs experienced by ≥ 2 participant or with the maximum severity category ≥ Grade 2 are included.

**TABLE 3:** Summary of adverse events of BRII-198-001 study.

	BRII-198 750 mg (n = 3)	BRII-198 1500 mg (n = 6)	BRII-198 3,000 mg (n = 3)	Placebo (n = 4)
Participants with any TEAE	3 (100%)	5 (83%)	0	4 (100%)
Participants with Grade 1 TEAE	2 (67%)	5 (83%)	0	3 (75%)
Participants with Grade 2 TEAE	1 (33%)	0	0	1 (25%)
Participants with Grade 3 or above TEAE	0	2^a^ (33%)	0	1^b^ (25%)
Participants with serious TEAE	0	0	0	1^b^ (25%)
Participants with infusion reaction TEAE	0	0	0	0
Participants with TEAEs related to study drug	2 (67%)	1 (17%)	0	0
Participants with any TEAE leading to discontinuation of study drug	0	0	0	0
Death	0	0	0	0
Number of participants with AEs by SOC^c^				
Investigations	3 (100%)	4 (67%)	0	3 (75%)
Gastrointestinal disorders	1 (33%)	1 (17%)	0	0
Injury, poisoning, and procedural complications	0	0	0	1 (25%)

Abbreviations: TEAE, treatment-emergent adverse event; SOC, system organ class. Participants who experienced the same AE on more than one occasion (based on the specific category) are counted once in each relevant category. Percentages are based on the number of participants in the treatment group.

a 1 participant had an elevated blood creatine phosphokinase of Grade 4 after excessive exercise, and 1 participant had increased blood triglycerides of Grade 3 on day 181. Both events were not considered as related to the study drug by the investigators and normalized within 1-3 weeks.

b 1 participant receiving placebo had a serious TEAE (bilateral traumatic foot fracture leading to hospitalization, which was also reported as a Grade 3 TEAE). The participant recovered after surgery.

c Only SOCs experienced by ≥ 2 participant or with the maximum severity category ≥ Grade 2 are included.

**TABLE 4:** Summary of adverse events of BRII-196-198-001 study.

	BRII-196/198 750/750 mg (n = 3)	BRII-196/198 1500/1500 mg (n = 6)	Placebo (n = 3)
Participants with any TEAE	3 (100)	5 (83.3)	2 (66.7)
Participants with grade 1 TEAE	2 (66.7)	5 (83.3)	1 (33.3)
Participants with grade 2 TEAE	2 (66.7)	1 (16.7)	2 (66.7)
Participants with grade 3 or above TEAE	1 (33.3)	0	0
Participants with serious TEAE	0	0	0
Participants with infusion reaction TEAE	0	0	0
Participants with TEAEs related to study drug	0	1 (16.7%)	0
Participants with any TEAE leading to discontinuation of study drug	0	0	0
Death	0	0	0
Number of participants with AEs by SOC^a^			
Investigations	3 (100%)	5 (83.3%)	2 (66.7%)

Abbreviations: TEAE, treatment-emergent adverse event; SOC, system organ class. Participants who experienced the same AE on more than one occasion (based on the specific category) are counted once in each relevant category. Percentages are based on the number of participants in the treatment group.

a Only SOCs experienced by ≥ 2 participant or with the maximum severity category ≥ Grade 2 are included.

### Pharmacokinetics

**FIGURE 1:**
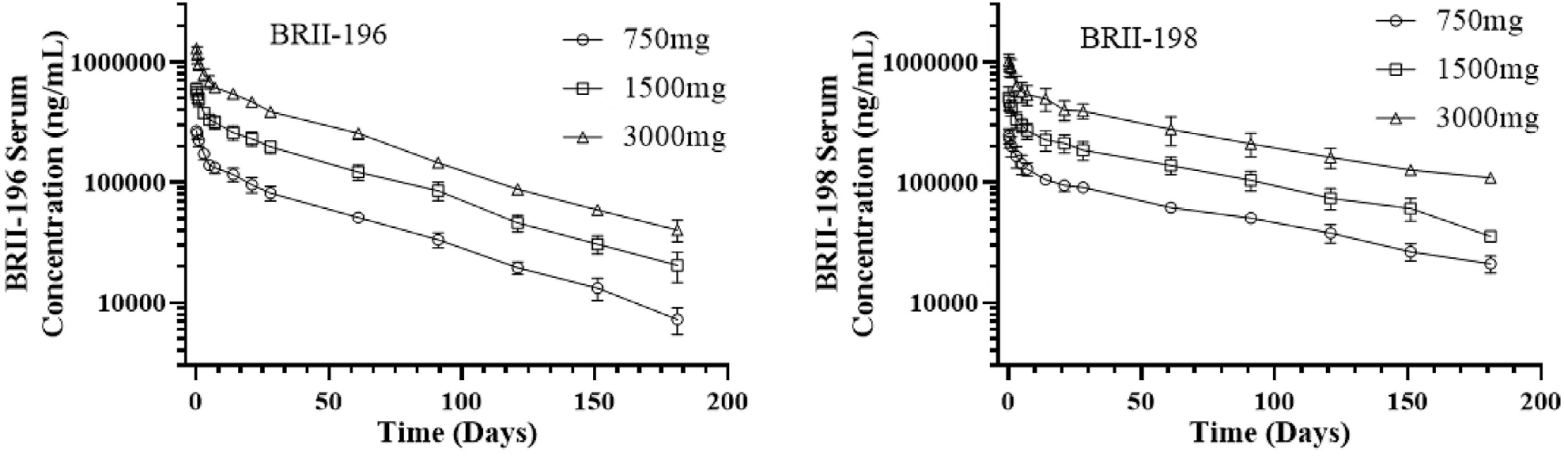
Observed mean (±Standard deviation) serum concentration - time profiles of BRII-196 and BRII-198 following a single intravenous infusion administration to the healthy adult participants.

Pharmacokinetic data were available for 33 participants, including 12 participants each for BRII-196 and BRII-198 alone, and 9 for BRII-196/BRII-198 combination over the 181-days study duration. Following a single IV infusion of BRII-196 or BRII-198 at 750, 1500, or 3,000 mg, mean serum PK parameters, including C_max_ and AUC, increased in an approximately dose-proportional manner (Table 5; Figure 1). The mean systemic serum clearance was 75.4 ml/day for BRII-196 and 57.0 ml/day for BRII-198. The mean terminal half-life (t_1/2_) was 44.6–48.6 days for BRII-196 and 72.2–83.0 days for BRII-198. The relatively shorter terminal half-life of BRII-196 correlated with a slightly higher systemic clearance. PK measurements of BRII-196 and BRII-198 among participants receiving the combination were highly consistent with those from single mAb studies (Supplementary Table S5), indicating no meaningful interactions between these two antibodies.

**TABLE 5:** BRII-196 and BRII-198 pharmacokinetic parameters following a single intravenous infusion administration to the healthy adult participants.

Antibody	Dose (mg)	Number of subject	C_max_ (μg/ml)	T_max_ (hour)	t_1/2_ (day)	CL (ml/day)	V_ss_ (L)	AUC_last_ (day× μg/mL)	AUC_inf_ (day× μg/mL)
BRII-196	750	3	268 (19.9)	4.6	48.6 (4.57)	76.8 (11.2)	4.86 (0.563)	8,240 (764)	8,950 (1,040)
	1500	6	598 (77.1)	5.4	46.0 (8.11)	72.3 (10.0)	4.50 (0.567)	19,300 (2,430)	21,100 (2,900)
	3,000	3	1300 (91.7)	6.6	44.7 (6.76)	72.0 (3.97)	4.37 (0.298)	38,900 (2,510)	41,700 (2,200)
BRII-198	750	3	244 (35.5)	4.7	76.0 (8.99)	59.4 (5.54)	6.19 (0.746)	10,400 (911)	12,800 (1,240)
	1500	6	509 (107)	5.4	72.3 (8.46)	57.0 (9.46)	5.78 (0.983)	20,900 (2,950)	26,900 (4,720)
	3,000	3	1020 (149)	6.8	83.2 (37.7)	53.8 (8.82)	6.08 (2.19)	42,800 (9,680)	56,700 (8,710)

Abbreviations: SD; standard deviation; C_max_, observed maximum serum concentration; T_max_, time to reach observed maximum serum concentration; t_1/2_, terminal half-life; CL, systemic clearance; V_ss_, volume of distribution at steady state; AUC_last_, area under the concentration–time curve from time zero to the last measurable concentration; AUC_inf_, area under the concentration–time curve from time zero to infinity. All parameters are reported with mean and standard deviation in three significant figures except T_max_ that is reported with median values.

### Anti-drug antibodies Response

In the BRII-196-001 study, 4 out of 12 participants tested positive for ADA in the screening assays, but all tested negative in the follow-up confirmatory assays. In the BRII-198-001 study, one participant who received BRII-198 had positive ADA samples in the screening assay but later were confirmed to be negative in the tier-2 confirmatory assay. All 9 participants receiving BRII-196/BRII-198 combination were tested negative for ADA both at baseline and throughout study follow-up.

### *In Vitro* Neutralization activities

**FIGURE 2:**
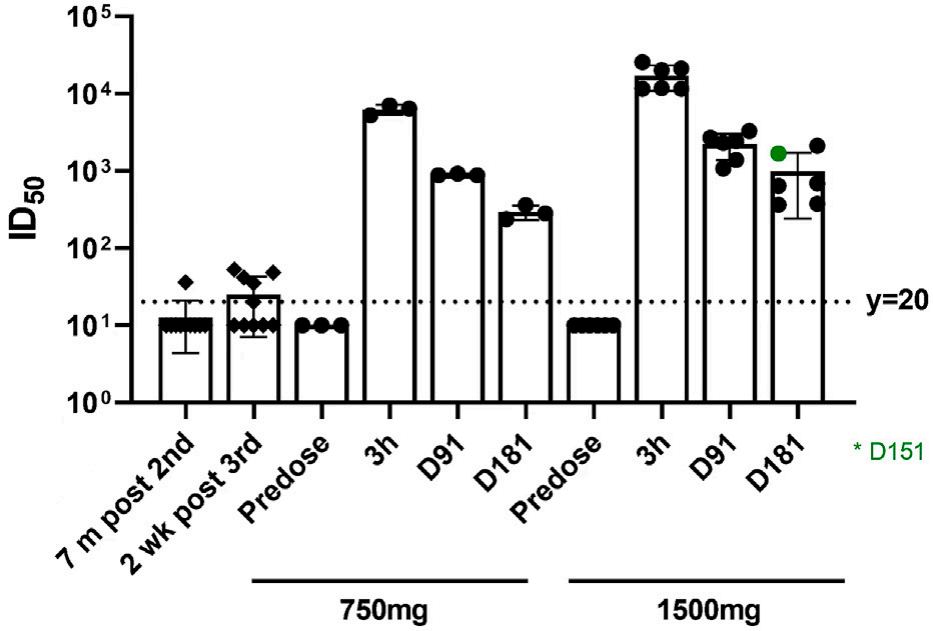
Neutralization activities Against the SARS-CoV-2 delta variant. The dotted line indicates the limit of detection of 20-fold dilution. The results below this limit were set to 10 for visualization.

The plasma neutralizing activity against live SARS-CoV-2 Delta variant were dynamically monitored up to 6 months post single injection of BRII-196/BRII-198 and compared with plasma of donors who received two or three doses of inactivated vaccines, as summarized in Figure 2. The median ID_50_ values of plasma taken 6 months after a single dose of BRII-196/BRII-198 at two dose levels, 750/750 mg and 1500/1500 mg, were 280.0 and 665.5 respectively, in comparison to 15.1 for plasma taken 2 weeks post the third dose of vaccine, indicating sufficient plasma PK exposures up to 6 months post single dose of BRII-196/BRII-198 IV administration to neutralize Delta variant.

## Discussion

The mAbs targeting specific viral epitopes, including BRII-196 and BRII-198, belong to a platform technology associated with a favorable safety profile. In our studies, intravenous administration of BRII-196 and BRII-198 was safe and well-tolerated in healthy adult participants with a single mAb dose up to 3,000 mg and in combination at 1500/1500 mg, with no infusion reactions or AEs leading to dose adjustment or discontinuation. Most AEs reported were grade 1-2 in severity, with the majority being isolated asymptomatic transient laboratory abnormalities. The safety observations in these phase 1 studies are reinforced by recent findings from phase 2 and phase 3 studies in which BRII-196 and BRII-198 combination at 1000 mg/1000 mg were not associated with clinically significant safety signals in comparison to placebo arms among both outpatients and hospitalized patients with COVID-19 (Evering et al., 2021; Self et al., 2022). In addition, as the global pandemic of SARS-CoV-2 is still evolving, the reported phase 1 studies provide supportive evidence when higher doses of BRII-196 and BRII-198 are considered.

With YTE modification, both BRII-196 and BRII-198 demonstrated anticipated 2-3 folds half-life extension in healthy adult participants. In comparison to BRII-198, a relatively shorter half-life was observed with BRII-196, which correlates with slightly higher systemic clearance. As both antibodies showed comparable increased binding affinities to human FcRns at pH 6.0 *in vitro* (Ji et al., submitted), different molecular properties such as charge variants, glycan profiles, and overall thermal stability might contribute to the observed differences. In addition, the pharmacokinetic profiles of BRII-196 and BRII-198, when used in combination, were consistent with those observed as monotherapies, suggesting there are no interactions between the two mAbs.

The prolonged half-life of BRII-196 and BRII-198 can be translated to a stable drug concentration during the treatment window. Additionally, this feature supports the use of the combination as an alternative to vaccination for pre-exposure prevention, representing a significant unmet need globally. For instance, it is estimated that 2.7% of the population are immunocompromised (Harpaz et al., 2016) and would not be adequately protected by COVID-19 vaccines. As the measurement of humoral immunity against SARS-CoV-2, neutralization activities conferred by antibodies were highly predictive of immune protection from symptomatic SARS-CoV-2 infection (Khoury et al., 2021). In this study, it is notable that higher neutralization activities in the plasma conferred by BRII-196/BRII-198 after 6 months of administration were considerably higher than that associated with plasma obtained 2 weeks post booster vaccination of BBIBP-CorV inactivated vaccine, suggesting robust and prolonged protective effect which can be confirmed in future clinical trials.

Limitations of this study include the small sample size of typical phase 1 studies, which prevented meaningful subgroup analyses by age group or gender. However, phase 3 studies did not identify clinically significant safety signals of BRII-196 and BRII-198 combination in comparison to placebo, which suggested an overall favorable safety profile of this antibody combination (Evering et al., 2021; Self et al., 2022). Second, only live Delta variant was included for the evaluation of neutralizing activities, which is not the current dominant variant for the global epidemic. As the evaluation in this report proved the concept that long acting monoclonal antibodies as BRII-196 and BRII-198 can render lasting humeral immune protection in comparison to certain vaccines, further researches are warranted to verify the findings on other existing and emerging variants including Omicron BA 4/5.

## Conclusion

In summary, BRII-196 and BRII-198 were safe and well-tolerated in healthy adult participants. Pharmacokinetic assessments demonstrated linear pharmacokinetics with prolonged half-lives that are associated with neutralization activity against SARS-CoV-2 Delta live virus *ex-vivo* for BRII-196/BRII-198 combination up to 180 days post a single infusion. Based on interim analysis from a platform phase 2/3 clinical study (ACTIV-2), BRII 196/BRII 198 at 1000mg/1000 mg were highly effective in reducing the risk of hospitalization or death by 78% in outpatients with mild to moderate COVID-19 (Evering et al., 2021). Taken together, these findings support further development and utility of BRII-196 and BRII-198 for the treatment and prophylaxis of SARS-CoV-2 infection to address unmet need in the pandemic.

## Data Availability

The original contributions presented in the study are included in the article/Supplementary Material, further inquiries can be directed to the corresponding authors.
