# Supplementary material for: Randomized, placebo-controlled, single-blind phase 1 studies of the safety, tolerability, and pharmacokinetics of BRII-196 and BRII-198, SARS-CoV-2 spike-targeting monoclonal antibodies with an extended half-life in healthy adults
Source: Front Pharmacol. 2022 Sep 6;13:983505. doi: 10.3389/fphar.2022.983505 (PMC9486188; doi:10.3389/fphar.2022.983505)
Supplement: Supplementary file 1 [file DataSheet1.docx]

Supplementary Appendix

**Section S1**

1. Numbers of Ethical Committee Approval

BRII-196-001 Study (2020-025-01; 2020-025-02)

BRII-198-001 Study (2020-026-02)

BRII-196-198-001 Study (2020-023-02; 2020-023-03; 2020-023-04; 2020-023-06; 2020-023-09)

**Section S2**

**Figure S1. Subject Disposition of Study BRII-196-001**


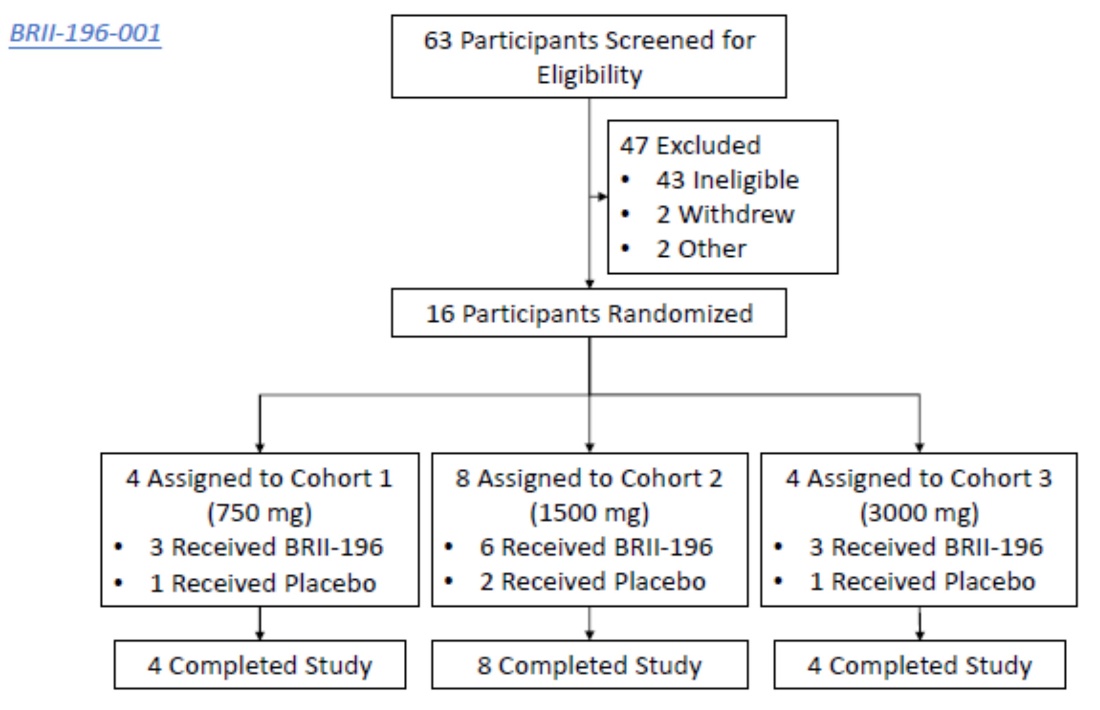


**Figure S2. Subject Disposition of Study BRII-198-001**


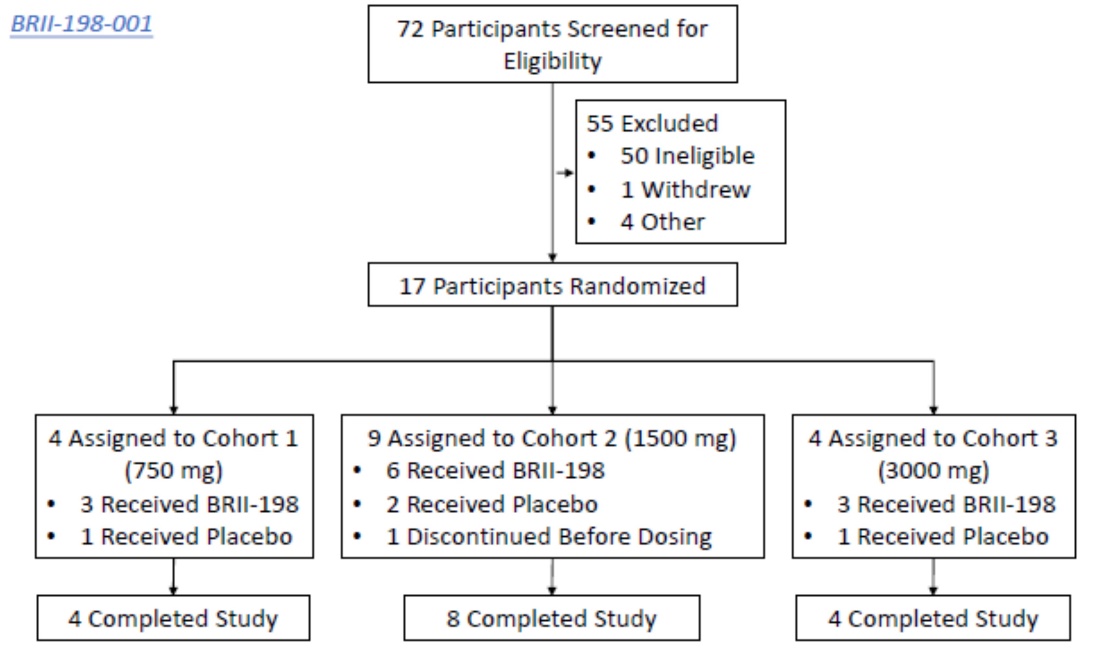


**Figure S3. Subject Disposition of Study BRII-196-198-001**


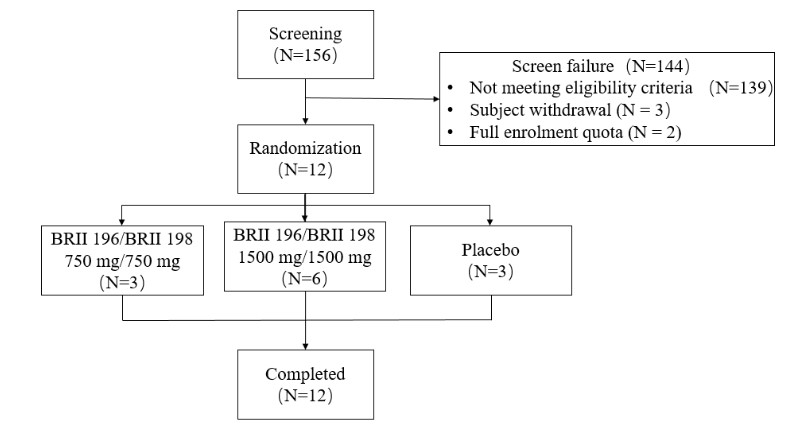


**Table S1. Adverse Events by System Organ Class and Preferred Term in BRII-196-001 and BRII-198-001 Studies**

| **Antibody** | **System Organ Class**  **Preferred Term** | **750 mg (n=3)** | **1500 mg (n=6)** | **3000 mg (n=3)** | **Placebo (n=4)** |
| --- | --- | --- | --- | --- | --- |
| **BRII-196** | Investigations | 3 (100%) | 4 (67%) | 2 (67%) | 4 (100%) |
|  | White blood cell count decreased | 1 (33%) | 3 (50%) | 0 | 1 (25%) |
|  | Blood triglycerides increased | 1 (33%) | 0 | 1 (33%) | 2 (50%) |
|  | Neutrophil count decreased | 1 (33%) | 2 (33%) | 0 | 1 (25%) |
|  | Blood uric acid increased | 1 (33%) | 1 (17%) | 1 (33%) | 0 |
|  | Alanine aminotransferase increased | 1 (33%) | 0 | 0 | 1 (25%) |
|  | Blood bilirubin increased | 0 | 1(17%) | 1 (33%) | 0 |
|  | Low density lipoprotein increased | 0 | 0 | 0 | 1 (25%) |
|  | Lymphocyte percentage decreased | 0 | 1 (17%) | 0 | 0 |
|  | Monocyte count decreased | 0 | 0 | 0 | 1 (25%) |
|  | Neutrophil count increased | 0 | 1 (17%) | 0 | 0 |
|  | Platelet count increased | 1 (33%) | 0 | 0 | 0 |
|  | White blood cells urine positive | 0 | 0 | 0 | 1 (25%) |
|  | Infections and infestations | 1 (33%) | 0 | 0 | 2 (50%) |
|  | Nasopharyngitis | 1 (33%) | 0 | 0 | 1 (25%) |
|  | Periodontitis | 1 (33%) | 0 | 0 | 0 |
|  | Tooth infection | 0 | 0 | 0 | 1 (25%) |
|  | Gastrointestinal disorders | 0 | 1 (17%) | 0 | 0 |
|  | Diarrhoea | 0 | 1 (17%) | 0 | 0 |
|  | Musculoskeletal and connective tissue disorders | 0 | 1 (17%) | 0 | 0 |
|  | Pain in extremity | 0 | 1 (17%) | 0 | 0 |
| **BRII-198** | Investigations | 3 (100%) | 4 (67%) | 0 | 3 (75%) |
|  | Blood triglycerides increased | 0 | 4 (67%) | 0 | 2 (50%) |
|  | Blood bilirubin increased | 1 (33%) | 2 (33%) | 0 | 0 |
|  | White blood cell count decreased | 1 (33%) | 1 (17%) | 0 | 1 (25%) |
|  | Alanine aminotransferase increased | 1 (33%) | 1 (17%) | 0 | 0 |
|  | Blood creatine phosphokinase increased | 0 | 1 (17%) | 0 | 0 |
|  | Blood uric acid increased | 0 | 0 | 0 | 1 (25%) |
|  | Aspartate aminotransferase increased | 0 | 1 (17%) | 0 | 0 |
|  | Injury, poisoning and procedural complications | 0 | 0 | 0 | 1 (25%) |
|  | Foot fracture | 0 | 0 | 0 | 1 (25%) |
|  | Ear and labyrinth disorders | 0 | 0 | 0 | 1 (25%) |
|  | Cerumen impaction | 0 | 0 | 0 | 1 (25%) |
|  | Gastrointestinal disorders | 1 (33%) | 1 (17%) | 0 | 0 |
|  | Abdominal pain upper | 1 (33%) | 0 | 0 | 0 |
|  | Constipation | 0 | 1 (17%) | 0 | 0 |
|  | Infections and infestations | 0 | 0 | 0 | 1 (25%) |
|  | Otitis externa | 0 | 0 | 0 | 1 (25%) |
|  | Musculoskeletal and connective tissue disorders | 0 | 0 | 0 | 1 (25%) |
|  | Arthralgia | 0 | 0 | 0 | 1 (25%) |

Participants who experienced the same AE on more than one occasion (based on the specific category) are counted once in each relevant category. Percentages are based on the number of participants in the treatment group.

**Table S2. Treatment Emergent Adverse Events by System Organ Class and Preferred Term in BRII-196-198-001 Study**

| **System Organ Class**  **Preferred Term** | **BRII-196/BRII-198** **750 mg/750 mg** **(N=3)** **n (%)** | **BRII-196/BRII-198** **1500 mg/1500 mg** **(N=6)** **n (%)** | **Placebo** **(N=3)** **n (%)** |
| --- | --- | --- | --- |
| Investigations | 3 (100%) | 5 (83%) | 2 (67%) |
| White blood cell count decreased | 1 (33%) | 1 (17%) | 1 (33%) |
| Blood bilirubin increased | 1 (33%) | 1 (17%) | 0 |
| Blood creatine phosphokinase increased | 1 (33%) | 1 (17%) | 0 |
| Blood triglycerides increased | 1 (33%) | 1 (17%) | 0 |
| Lymphocyte count decreased | 0 | 1 (17%) | 1 (33%) |
| Red blood cells urine positive | 0 | 2 (33%) | 0 |
| Blood uric acid increased | 0 | 1 (17%) | 0 |
| Blood pressure increased | 1 (33%) | 0 | 0 |
| Hemoglobin decreased | 0 | 0 | 1 (33%) |
| Cardiac disorders | 1 (33%) | 1 (17%) | 0 |
| Sinus bradycardia | 1 (33%) | 0 | 0 |
| Ventricular extrasystoles | 0 | 1 (17%) | 0 |

**Table S3. Treatment-related Adverse Events by Preferred Term in BRII-196-001 and BRII-198-001 Studies**

| **Antibody** | **Preferred Term** | **750 mg (n=3)** | **1500 mg (n=6)** | **3000 mg (n=3)** | **Placebo (n=4)** |
| --- | --- | --- | --- | --- | --- |
| **BRII-196** | White blood cell count decreased | 0 | 1 (17%) | 0 | 0 |
|  | Neutrophil count decreased | 0 | 1 (17%) | 0 | 0 |
|  | Alanine aminotransferase increased | 1 (33%) | 0 | 0 | 0 |
|  | Blood bilirubin increased | 0 | 1 (17%) | 0 | 0 |
| **BRII-198** | Alanine aminotransferase increased | 1 (33%) | 0 | 0 | 0 |
|  | Blood bilirubin increased | 1 (33%) | 1 (17%) | 0 | 0 |

Participants who experienced the same AE on more than one occasion (based on the specific category) are counted once in each relevant category. Percentages are based on the number of participants in the treatment group.

**Table S4. Treatment-related Adverse Events by Preferred Term in BRII-196-198-001 Study**

| **Preferred Term** | **BRII-196/BRII-198** **750 mg/750 mg** **(N=3)** **n (%)** | **BRII-196/BRII-198** **1500 mg/1500 mg** **(N=6)** **n (%)** | **Placebo** **(N=3)** **n (%)** |
| --- | --- | --- | --- |
| White blood cell count decreased | 0 | 1 (17%) | 0 |
| Lymphocyte count decreased | 0 | 1 (17%) | 0 |

Participants who experienced the same AE on more than one occasion (based on the specific category) are counted once in each relevant category. Percentages are based on the number of participants in the treatment group.

**Table S5. BRII-196 and BRII-198 Pharmacokinetic Parameters Following Subsequent Intravenous Infusion to Healthy Adult Participants**

| Antibody | Dose  (mg) | Number of Subject | C_max_  (mg/mL) | T_max_  (hour) | t_1/2_  (day) | CL  (mL/day) | V_ss_  (L) | AUC_last_  (day×mg/mL) | AUC_inf_  (day×mg/mL) |
| --- | --- | --- | --- | --- | --- | --- | --- | --- | --- |
| BRII-196 | BRII-196 /BRII-198 750/750 | 3 | 310 (55.0) | 4.0 | 47.1 (3.37) | 75.1 (9.96) | 4.86 (0.829) | 9360 (1400) | 10100 (1440) |
|  | BRII-196 /BRII-198 1500/1500 | 6 | 607 (107) | 4.1 | 51.0 (11.2) | 75.8 (13.9) | 5.22 (0.861) | 18200 (2940) | 20300 (3540) |
| BRII-198 | BRII-196 /BRII-198 750/750 | 3 | 245 (29.4) | 9.0 | 71.2 (9.21) | 61.7 (9.58) | 6.21 (0.484) | 10100 (1220) | 12300 (1920) |
|  | BRII-196 /BRII-198 1500/1500 | 6 | 431 (51.9) | 4.0 | 77.6 (11.3) | 66.5 (14.8) | 7.00 (0.973) | 18500 (3140) | 23500 (5030) |

C_max_=observed maximum serum concentration, T_max_=time to reach observed maximum serum concentration, t_1/2_=terminal half-life, CL=systemic clearance, V_ss_=volume of distribution at steady state, AUC_last_=area under the concentration–time curve from time zero to the last measurable concentration, AUC_inf_=area under the concentration–time curve from time zero to infinity. All parameters are reported with mean and standard deviation in three significant figures except T_max_ that is reported with median values.
